# Resilience and related factors in alcohol use disorder: the role of trauma, impulsivity, aggression, and temperament

**DOI:** 10.3389/fpsyg.2025.1718314

**Published:** 2025-12-05

**Authors:** Naile Özge Utkan, Güliz Şenormancı, Çetin Turan, Salih Metin, Ömer Şenormancı

**Affiliations:** 1 Department of Psychiatry, University of Health Sciences Bursa City Hospital, Bursa, Türkiye; 2 Department of Psychiatry, University of Health Sciences Bursa Yuksek Ihtisas Training and Research Hospital Kurtuluş, Bursa, Türkiye; 3 Department of Family Medicine, University of Health Sciences Bursa City Hospital, Bursa, Türkiye

**Keywords:** alcohol use disorder, resilience, impulsivity, temperament, childhood trauma

## Abstract

**Background:**

Resilience—the capacity to adapt effectively to stress and adversity—plays a protective role across psychiatric disorders. Individuals with alcohol use disorder (AUD) often present with lower resilience, potentially influenced by impulsivity, aggression, temperament, and childhood trauma. This study examined how these factors relate to resilience in individuals with AUD.

**Methods:**

Between September 2024 and March 2025, 74 male inpatients with DSM-5-TR–diagnosed AUD were recruited from the University of Health Sciences Bursa Yuksek Ihtisas Training and Research Hospital. Participants completed the Resilience Scale for Adults (RSA), Beck Depression Inventory (BDI), Barratt Impulsiveness Scale-11 (BIS-11), Buss–Perry Aggression Questionnaire (AQ), Temperament Evaluation of Memphis, Pisa, Paris, and San Diego Autoquestionnaire (TEMPS-A), and the Childhood Trauma Questionnaire (CTQ). Correlation and regression analyses were conducted.

**Results:**

Higher RSA scores were observed among employed participants and among those without a history of suicide attempts. Depression scores showed weak negative correlations with RSA subscales, particularly social competence and social resources. Impulsivity—especially non-planning impulsivity—showed moderate-to-strong negative associations with resilience and emerged as its strongest predictor. Depressive and irritable temperaments were significant predictors of lower resilience, whereas hyperthymic temperament was weakly positively associated. Childhood emotional abuse and neglect showed a weak but significant negative correlation with resilience—particularly with perception of future—while physical and sexual abuse were not significantly related. Aggression showed no consistent associations with resilience, apart from a weak negative correlation between hostility and perception of future. The final regression model (non-planning impulsivity, depressive and irritable temperaments) explained 35.8% of the variance in resilience.

**Conclusion:**

In AUD, resilience is negatively associated with depressive and irritable temperaments and with non-planning impulsivity, and is modestly related to childhood emotional maltreatment. Although shaped by relatively stable traits and early adverse experiences, resilience may be enhanced through psychosocial interventions—particularly those targeting emotion regulation, future orientation, and social support—which could help improve clinical outcomes in AUD.

## 1 Introduction

Studies showed that some individuals were able to develop healthily despite adverse life circumstances, leading to the growing importance of the concept of “resilience” in psychology and psychiatry (Fadardi et al., 2010). Resilience is defined as the capacity to maintain stability, recover quickly, and even grow stronger after significant adverse events (Leipold and Greve, 2009). It represents an active adaptation process shaped by the interaction of individual characteristics, family structure, social support, and environmental factors (Fletcher and Sarkar, 2013).

The protective role of resilience has been widely studied in post-traumatic stress disorder, depression, schizophrenia, bipolar disorder, and substance use disorders. These studies indicate that individuals with mental disorders generally show lower resilience, while higher resilience may prevent illness onset or reduce symptom severity (Agaibi and Wilson, 2005; Kesebir et al., 2013; Şenormancı et al., 2020; Şenormanci et al., 2022; Yamashita et al., 2021). Therefore, resilience is increasingly considered a core protective factor across various psychiatric conditions.

Alcohol use disorder (AUD) is a chronic and recurrent condition characterized by impaired control over alcohol intake, compulsive use, and withdrawal symptoms (World Health Organization, 2018). It is especially common in high- and upper-middle-income countries (Rehm and Shield, 2019). Recent studies have reported that higher resilience is associated with lower alcohol use, reduced severity of dependence, and decreased relapse risk (Cusack et al., 2023).

The concept of resilience historically emerged from research on adverse childhood experiences (Herrman et al., 2011). Severe and repeated childhood stressors may have lasting consequences on both physical and mental health (Smith and Pollak, 2020). Childhood trauma has been identified as an important risk factor for the development of AUD (Kendler et al., 2000; Schuck and Widom, 2001), and has been associated with more severe symptoms and poorer treatment outcomes in alcohol users (Kesebir et al., 2015; Masten and Tellegen, 2012). At the same time, some individuals develop coping mechanisms that enhance resilience despite such experiences (Sordi et al., 2015). Emotional abuse and neglect in childhood, however, have consistently been linked to lower resilience (Wingo et al., 2014).

Low resilience to stress may also increase vulnerability to psychiatric conditions such as depressive disorders (Bogdan et al., 2013). Depression, particularly major depressive disorder, is one of the most common comorbidities in AUD (Grant et al., 2004), and this comorbidity is associated with worse clinical outcomes and prognosis (Hasin et al., 2002). Individuals with depression have been shown to exhibit significantly lower resilience and subscale scores compared to healthy controls (He et al., 2022; Kesebir et al., 2013; Paul and Panda, 2025).

Impulsivity, defined as a tendency to act quickly and without planning in response to internal or external stimuli, is another relevant factor (Moeller et al., 2001). Alcohol use and related problems are strongly associated with impulsivity (Dick et al., 2010; Jakubczyk et al., 2018), which plays a central role in the initiation, maintenance, and relapse of addictive behaviors (Koob, 2011; Sliedrecht et al., 2019). Studies suggest that individuals with low resilience exhibit higher impulsivity, which in turn is linked to problematic alcohol use (Meyer et al., 2025; Şenormanci et al., 2025).

Aggression is also frequently observed in individuals with AUD, particularly during intoxication and withdrawal periods (Heinrichs, 1989; Heinz et al., 2011; Martin et al., 2019; Mirijello et al., 2015). Alcohol impairs prefrontal cortical functioning, reduces cognitive control, and facilitates aggressive behaviors (Heinrichs, 1989). Research has demonstrated that patients with AUD show higher aggression, while resilience is negatively associated with especially physical aggression (Ispir et al., 2025; Pachi et al., 2021).

In recent years, temperament and character traits have been increasingly studied in relation to substance use disorders. Patients with AUD tend to score higher on depressive, cyclothymic, and irritable temperaments (Pacini et al., 2009). They also exhibit higher novelty seeking and harm avoidance, along with lower self-directedness (Avila Escribano et al., 2016). According to Cloninger’s biopsychosocial model, temperament determines automatic emotional responses to environmental stressors and can directly influence resilience (Kim et al., 2013). High harm avoidance and low self-directedness have been linked to maladaptive coping strategies and lower psychological resilience (Cloninger et al., 1994). Such temperament patterns may facilitate reliance on external regulatory mechanisms, such as alcohol use (Wills and Hirky, 1996). Although Cloninger’s model provides a broader biopsychosocial framework for temperament, the present study employed the Temperament Evaluation of Memphis, Pisa, Paris, and San Diego Autoquestionnaire (TEMPS-A) (Akiskal et al., 2005), as it specifically measures affective temperaments (depressive, cyclothymic, irritable, hyperthymic, and anxious), which have been shown to be more directly associated with resilience and addiction-related behaviors.

According to resilience theories proposed by Masten (2001) and Rutter (1987), resilience is not simply the absence of psychopathology but a dynamic process of positive adaptation despite significant adversity. From this perspective, childhood trauma and temperament traits constitute vulnerability factors, whereas resilience acts as a protective factor that may buffer their impact on the development and severity of AUD. Recent work suggests that resilience may also contribute to relapse processes and treatment adherence in AUD; however, its precise role remains less clearly defined than that of established correlates such as impulsivity, depression, and trauma (Cusack et al., 2023; Sliedrecht et al., 2019).

The aim of this study was to investigate how resilience is associated with depressive and irritable temperaments, impulsivity traits, and childhood emotional maltreatment in individuals with AUD. We hypothesized that lower resilience would be significantly associated with higher levels of childhood emotional maltreatment and impulsivity, and that depressive and irritable temperament traits would predict lower resilience.

## 2 Methods

### 2.1 Participants

Between September 2024 and March 2025, a total of 74 male inpatients aged 18–50 years, diagnosed with alcohol use disorder (AUD) according to DSM-5-TR criteria, were recruited from the Research, Treatment and Training Center for Alcohol and Substance Dependence, University of Health Sciences Bursa Yuksek Ihtisas Training and Research Hospital.

Inclusion criteria were hospitalization at the center with a primary diagnosis of AUD. Exclusion criteria included neurocognitive disorders, intellectual disability, severe psychiatric disorders (e.g., schizophrenia, bipolar disorder), history of other substance use disorders, or illiteracy. All participants underwent a clinical evaluation to confirm the primary diagnosis of AUD.

The following instruments were administered: the Resilience Scale for Adults (RSA), Beck Depression Inventory (BDI), Barratt Impulsiveness Scale-11 (BIS-11), Buss-Perry Aggression Questionnaire (AQ), Temperament Evaluation of Memphis, Pisa, Paris and San Diego Autoquestionnaire (TEMPS-A), and the Childhood Trauma Questionnaire (CTQ). Sociodemographic and clinical data were collected through semi-structured interviews and review of medical records.

To estimate the required sample size, data from the first 30 participants were analyzed. The lowest correlation coefficient between resilience and other measures was 0.33. Based on this effect size, with *α* = 0.05 and power = 0.80, a minimum of 70 participants was determined to be sufficient.

Alcohol consumption was calculated in terms of standard drinks. Rakı, whiskey, gin, brandy, and vodka were considered to contain similar alcohol levels, with 70 cL corresponding to approximately 30 units. Beer (0.33 L) and wine (0.15 L) were recorded as one unit each, based on self-reports.

All participants were informed about the study and provided written consent in accordance with the Declaration of Helsinki. The study protocol was approved by the Ethics Committee of the University of Health Sciences (Approval No: 2011-KAEK-252021/02-09).

### 2.2 Measures

#### 2.2.1 Sociodemographic data form

Sociodemographic data including age, marital status, level of education, employment status, socioeconomic status, smoking status, duration of alcohol use, number of previous quit attempts, previous alcohol-related inpatient treatments, place of residence, living arrangement, number of children, history of suicide attempts, psychiatric medication use, and presence of other medical conditions were collected through structured interviews and medical records.

#### 2.2.2 Beck Depression Inventory (BDI)

Depressive symptoms were assessed using the BDI, a 21-item self-report scale developed to measure emotional, cognitive, somatic, and motivational aspects of depression (Beck et al., 1979). Each item is rated on a 4-point Likert-type scale (0–3), and total scores are obtained by summing all item scores. The Turkish adaptation of the scale was conducted by Hisli (1989), who confirmed its reliability with a Cronbach’s alpha of 0.80. The Turkish validation study also suggested a cut-off score of 17 for indicating clinically significant depressive symptoms.

#### 2.2.3 Resilience Scale for Adults (RSA)

Resilience was assessed using the RSA, a self-report instrument developed by Friborg et al. (2003) to evaluate protective factors associated with resilience. The scale consists of 33 items rated on a 5-point semantic differential scale and comprises six subscales: perception of self, perception of future, structured style, social competence, family cohesion, and social resources. It measures individuals’ personal strengths, planning ability, social adaptation, and perceived support from family and social environment. The Turkish adaptation was performed by Basim and Cetin (2011), who reported Cronbach’s alpha coefficients ranging from 0.66 to 0.81 across the subscales, indicating acceptable internal consistency.

#### 2.2.4 Barratt Impulsiveness Scale-11 (BIS-11)

Impulsivity was assessed using the BIS-11, a 30-item self-report instrument developed to evaluate the multidimensional structure of impulsivity (Patton et al., 1995). The scale consists of three subscales: attentional impulsivity (difficulty sustaining attention and rapid decision-making), motor impulsivity (acting without thinking, rapid behavioral responses), and non-planning impulsivity (lack of forethought and a failure to consider future consequences). Each item is scored on a 4-point Likert scale (1 = rarely/never to 4 = almost always/always), with higher scores indicating greater impulsivity. The Turkish adaptation was conducted by Güleç et al. (2008), who reported a Cronbach’s alpha of 0.80, indicating good internal consistency.

#### 2.2.5 Buss–Perry Aggression Questionnaire (AQ)

Aggression was measured using the AQ, a 29-item self-report instrument developed to assess different dimensions of aggressive behavior (Buss and Perry, 1992). The scale comprises four subscales: physical aggression, verbal aggression, anger, and hostility. Items are rated on a 5-point Likert scale (1 = extremely uncharacteristic of me to 5 = extremely characteristic of me), and higher scores indicate greater levels of aggression. The Turkish version of the scale was adapted and validated by Madran (2013), who reported a Cronbach’s alpha of 0.85 for the total scale, indicating high internal consistency.

#### 2.2.6 Temperament Evaluation of Memphis, Pisa, Paris, and San Diego Autoquestionnaire (TEMPS-A)

Affective temperament traits were assessed using the TEMPS-A, a self-report questionnaire developed to measure five temperament dimensions: depressive, cyclothymic, hyperthymic, irritable, and anxious temperaments (Akiskal et al., 2005). The Turkish version consists of 100 items, of which 99 are scored dichotomously (1 = “yes,” 0 = “no”) across the five subscales: depressive (18 items), cyclothymic (19 items), hyperthymic (20 items), irritable (18 items), and anxious (24 items) (Vahip et al., 2005). The Turkish validation study demonstrated good internal consistency, with Cronbach’s alpha coefficients ranging from 0.77 to 0.85 across subscales.

#### 2.2.7 Childhood Trauma Questionnaire (CTQ)

Childhood trauma was assessed using the CTQ, developed by Bernstein et al. (1994). The original scale consists of five subscales: emotional abuse, emotional neglect, physical abuse, physical neglect, and sexual abuse. In the Turkish validation study (Şar et al., 2012), all five subscales demonstrated acceptable internal consistency (*α* = 0.65–0.89). However, several Turkish studies have reported that emotional abuse and emotional neglect are highly correlated and do not clearly separate in factor analyses, while physical neglect often loads together with physical abuse or shows weak factor loading in Turkish samples. Therefore, to ensure statistical robustness and consistency with previous Turkish research, the present study categorized childhood trauma into three domains: emotional abuse/neglect (EA–EN), physical abuse including physical neglect (PA), and sexual abuse (SA).

#### 2.2.8 Michigan Alcoholism Screening Test (MAST)

Alcohol-related problems were assessed using the MAST, a widely used self-report instrument developed to identify problematic alcohol use and its social consequences (Gibbs, 1983). The scale consists of 25 yes/no questions evaluating alcohol-related difficulties, loss of control, social or legal consequences, and treatment-seeking behaviors. Higher scores indicate greater impairment related to alcohol use. The Turkish adaptation and validation were conducted by Coşkunol et al. (1995), who reported high reliability (Cronbach’s α = 0.79) and validity. A cut-off score between 5–9 has been suggested to optimally distinguish individuals with and without problematic alcohol use in Turkish clinical samples. Although the scale can be self-administered, it is recommended to be evaluated by a clinician in cases where denial or minimization of alcohol use is suspected.

### 2.3 Statistical analysis

All statistical analyses were performed using SPSS version 22. The normality of continuous variables was assessed using the Shapiro–Wilk test. Descriptive statistics were reported as mean ± standard deviation (SD) or median (minimum–maximum) for continuous variables, and as frequencies and percentages (%) for categorical variables.

For bivariate comparisons, independent-samples t-tests were used for normally distributed variables, whereas Mann–Whitney U tests were used for non-normally distributed variables. Pearson correlation analysis was applied to normally distributed data, and Spearman correlation analysis was used for non-normally distributed variables.

To determine independent predictors of RSA total scores, a multiple linear regression analysis (backward elimination method) was conducted. Variables included in the regression model were those that were both theoretically relevant and showed significant correlations with resilience: BIS-11 non-planning impulsivity, BIS-11 motor impulsivity, TEMPS-A depressive, TEMPS-A irritable, and CTQ emotional abuse–neglect. Although motor impulsivity showed weaker associations in bivariate analyses, it was included initially due to its conceptual relevance to behavioral dysregulation in addiction. Regression assumptions (normal distribution of residuals, linearity, homoscedasticity, and absence of multicollinearity) were checked and met.

The level of statistical significance was set at *p* < 0.05. Findings with *p* < 0.001 were additionally reported to emphasize results with stronger statistical significance and to improve interpretability. These thresholds were applied consistently across analyses and did not reflect different decision rules. Because the bivariate analyses were exploratory in nature, no correction for multiple comparisons (e.g., Bonferroni) was applied; however, effect sizes were calculated to enhance interpretability and reduce overreliance on *p*-values. Effect sizes were reported where applicable: Cohen’s d or eta-squared (η^2^) for independent-samples t-tests, and rank-biserial correlation (r = Z/√N) for Mann–Whitney U tests.

A *post hoc* power analysis was conducted using G*Power 3.1 for the final regression model. With three predictors, *α* = 0.05, an effect size of f^2^ = 0.56 (calculated from Adjusted *R*^2^ = 0.358), and a sample size of *n* = 74, the statistical power was found to be 0.93. This indicates that the study had sufficient power to detect medium-to-large effect sizes.

## 3 Results

The mean age of participants was 43.2 ± 10.3 years, and the median duration of education was 8 years (5–15). Twenty-nine participants (39.2%) were single/divorced, while 45 (60.8%) were married. Among the sample, 45.9% were employed and 54.1% unemployed. Regarding socioeconomic status, 31.1% were in the lower group and 68.9% in the middle/upper group. The mean duration of alcohol use was 23.4 ± 11.0 years. Demographic and clinical characteristics are presented in Table 1, and psychometric scale scores in Table 2.

**Table 1:** Sociodemographic and clinical characteristics of the participants (*n* = 74).

Variable	Value
Age (years)	43.2 ± 10.3
Years of education	8 (5–15)
Duration of alcohol use (years)	23.4 ± 11.0
Daily alcohol consumption (standard drinks)	20 (5–45)
Number of previous quit attempts	2 (0–23)
Number of previous alcohol-related inpatient treatments	0 (0–16)
Marital status	
Single/divorced	29 (39.2%)
Married	45 (60.8%)
Duration of marriage (years, if married)	19.9 ± 10.5
Number of children	2 (0–6)
Socioeconomic status	
Low	23 (31.1%)
Middle/high	51 (68.9%)
Employment status	
Employed	34 (45.9%)
Unemployed	40 (54.1%)
Place of residence	
Town/district	30 (40.5%)
City	44 (59.5%)
Living arrangement	
With spouse/children	45 (60.8%)
With parents	15 (20.3%)
Alone/with relatives	14 (18.9%)
Smoking status	63 (85.1%)
Psychiatric medication use	36 (48.6%)
History of suicide attempt	19 (25.7%)
History of alcohol-related legal problems	15 (20.3%)
Other medical conditions	17 (23%)

Values are presented as mean ± standard deviation (SD), median (minimum–maximum), or frequency (percentage), as appropriate.

**Table 2:** Scores obtained by participants from the measurement tools (*n* = 74).

Measure / subscale	Mean ± SD/Median (min–max)
BDI	15 (2–40)
RSA	
Perception of self	22 (6–30)
Perception of future	12 (4–20)
Structured style	12 (4–20)
Social competence	17.8 ± 5.9
Family cohesion	18.9 ± 5.8
Social resources	26 (11–35)
Total	105.5 ± 24.1
BIS-11	
Attent’ional impulsiveness	33.2 ± 5.8
Motor impulsiveness	15 (9–26)
Non-planning impulsiveness	23 (15–35)
Total	72 (46–106)
AQ	
Verbal aggression	8 (0–19)
Physical aggression	13 (3–33)
Anger	12.6 ± 6.0
Hostility 16	(3–29)
Total	52.6 ± 18.5
TEMPS-A	
Cyclothymic	15 (2–19)
Depressive	10 (0–16)
Irritable	8.5 (0–17)
Hyperthymic	10.7 ± 4.0
Anxious	14 (0–24)
CTQ	
Emotional abuse and neglect	39.5 (19–77)
Physical abuse	28 (16–61)
Sexual abuse	5 (5–23)
MAST	32 (7–51)

Values are presented as mean ± standard deviation (SD) or median (min–max), depending on data distribution. BDI, Beck Depression Inventory; RSA, Resilience Scale for Adults; BIS-11, Barratt Impulsiveness Scale; AQ, Aggression Questionnaire; TEMPS-A, Temperament Evaluation of Memphis, Pisa, Paris, and San Diego; CTQ, Childhood Trauma Questionnaire; MAST, Michigan Alcoholism Screening Test.

### 3.1 Associations of RSA with demographic and clinical variables

When RSA subscales were compared with sociodemographic and clinical variables (Table 1), employed participants scored significantly higher on family cohesion than unemployed participants (*t* = 2.458, df = 72, *p* = 0.016, η^2^ = 0.077). Participants without a history of suicide attempt scored higher on perception of self than those with such a history (*Z* = −2.641, *p* = 0.008, *r* = 0.31). No other significant associations were observed.

### 3.2 Associations of RSA with psychiatric scales

Correlation analyses showed weak negative correlations between depression (BDI) scores and RSA subscales, particularly with social competence (*r* = −0.31, *p* < 0.05) and social resources (*r* = −0.31, *p* < 0.05).

RSA scores were negatively associated with impulsivity (BIS-11). Correlation coefficients were reported in descending order of magnitude rather than using subjective terms such as “strongest.” Non-planning impulsivity (*r* = −0.44 to −0.48, *p* < 0.001) and attentional impulsivity (*r* = −0.45 to −0.50, *p* < 0.001) showed the strongest and most consistent negative associations with RSA subscales. Motor impulsivity demonstrated weaker and less consistent correlations (r ranging from −0.10 to −0.34), remaining significant only for selected subscales.

Associations between RSA and aggression (AQ) were generally weak. Only verbal aggression showed a small positive correlation with social competence and social resources (*r* = 0.25, *p* < 0.05). No other aggression subscales were significantly associated with RSA.

Temperament dimensions demonstrated moderate negative correlations with resilience. Specifically, cyclothymic (*r* = −0.49, *p* < 0.001), depressive (*r* = −0.51, *p* < 0.001), irritable (*r* = −0.45, *p* < 0.001), and anxious (*r* = −0.50, *p* < 0.001) temperaments were inversely associated with RSA scores. Hyperthymic temperament was not significantly related to resilience.

Childhood trauma (CTQ) scores showed a weak but significant negative correlation between emotional abuse/neglect (EA–EN) and RSA total score (*r* = −0.25, *p* < 0.05). Physical abuse and sexual abuse subscales were not significantly associated with resilience.

MAST scores, reflecting alcohol-related problem severity, were weakly negatively correlated with resilience, particularly with perception of self (*r* = −0.32, *p* < 0.05) and perception of future (*r* = −0.38, *p* < 0.05) (see Table 3).

**Table 3:** Correlations between RSA subscales and psychological variables (*n* = 74).

Variable	Perception of Self	Perception of Future	Structured Style	Social Competence	Family Cohesion	Social Resources	Total
BDI	−0.17	−0.20	−0.15	−0.31*	−0.04	−0.31*	−0.22
BIS-11							
Attentional impulsivity	−0.45**	−0.40**	−0.27*	−0.06+	−0.26*+	−0.05	−0.31*+
Motor impulsiveness	−0.34*	−0.21	−0.10	−0.34*	−0.09	−0.34*	−0.30*
Non-planning impulsivity	−0.48**	−0.45**	−0.31*	−0.22	−0.20	−0.22	−0.44**
Total	−0.50**	−0.47**	−0.27*	−0.18	−0.17	−0.18	−0.39**
AQ							
Verbal aggression	−0.05	−0.16	0.05	0.25*	0.08	0.25*	0.09
Physical aggression	−0.01	−0.48	−0.09	−0.04	−0.13	−0.04	−0.08
Anger	−0.13	−0.14	−0.09	−0.04	0.00	−0.04	−0.11
Hostility	−0.14	−0.27*	−0.13	−0.07	−0.13	−0.07	−0.22
Total	−0.10	−0.19	−0.07	−0.19^+^	−0.08^+^	0.02	−0.14^+^
TEMPS-A							
Cyclothymic	−0.49**	−0.49**	−0.31*	−0.06	−0.31*	−0.06	−0.41**
Depressive	−0.39*	−0.52**	−0.21	−0.22	−0.48*	−0.22	−0.51**
Irritable	−0.48**	−0.41**	−0.35*	−0.21	−0.33*	−0.21	−0.45**
Hyperthymic	0.08	0.09	−0.08	0.10^+^	0.22^+^	0.10	0.15^+^
Anxious	−0.36*	−0.50**	−0.23*	−0.03	−0.13	−0.03	−0.30*
CTQ							
EA–EN	−0.11	−0.32^*^	−0.01	−0.13	−0.22	−0.13	−0.25^*^
PA	−0.09	−0.33^*^	−0.04	0.00	−0.18	0.00	−0.19
SA	−0.08	−0.17	−0.11	−0.25^*^	0.02	−0.25^*^	−0.18
MAST	−0.32^*^	−0.38^*^	−0.19	0.14	−0.18	0.14	−0.18

^+^, Pearson correlation; no symbol, Spearman correlation, *p < 0.05, **p < 0.001.

RSA, Resilience Scale for Adults; BDI, Beck Depression Inventory; BIS-11, Barratt Impulsiveness Scale, 11th version; AQ, Buss–Perry Aggression Questionnaire; TEMPS-A, Temperament Evaluation of Memphis, Pisa, Paris and San Diego autoquestionnaire version; CTQ, Childhood Trauma Questionnaire; EA-EN, Emotional Abuse and Emotional Neglect; PA, Physical Abuse; SA, Sexual Abuse; MAST, Michigan Alcoholism Screening Test.

### 3.3 Predictors of RSA total score

To determine the predictors of resilience, a multiple linear regression analysis was conducted with RSA total score as the dependent variable. Independent variables were selected based on theoretical relevance and significant correlations with resilience: BIS-11 motor impulsivity, BIS-11 non-planning impulsivity, TEMPS-A depressive temperament, TEMPS-A irritable temperament, and childhood emotional abuse/neglect (CTQ EA–EN). Although motor impulsivity showed weaker correlations in bivariate analyses, it was included in the initial regression model due to its established role in behavioral dysregulation and relapse risk in alcohol use disorders.

The analysis was performed using the backward elimination method. During the stepwise procedure, CTQ EA–EN was excluded in the second step and BIS-11 motor impulsivity in the third step. The final model (Model 4) was statistically significant (*F* = 14.581, *p* < 0.001) and accounted for 35.8% of the variance in resilience (Adjusted *R*^2^ = 0.358). In this model, BIS-11 non-planning impulsivity negatively predicted resilience (*B* = −1.268, *β* = −0.251, *p* = 0.017), TEMPS-A depressive temperament was also a significant negative predictor (*B* = −1.884, *β* = −0.305, *p* = 0.006), and TEMPS-A irritable temperament independently predicted lower resilience (*B* = −1.228, *β* = −0.254, *p* = 0.025). Regression assumptions were met: residuals were normally distributed, homoscedasticity and linearity were visually confirmed, and no multicollinearity was detected (VIF values between 1.16 and 1.47) (Table 4).

**Table 4:** Predictors of RSA total score (*n* = 74).

Dependent variable	Independent variable	*B*	*p*-value
RSA total	Constant	162.869	<0.001**
	BIS-11 non-planning impulsivity	−1.268	0.017*
	TEMPS-A depressive	−1.884	0.006*
	TEMPS-A irritable	−1.228	0.025*

Model summary: *F* = 14.581, *p* < 0.001**, Adjusted *R*^2^ = 0.358.

Linear regression, backward elimination method. Table presents unstandardized coefficients (*B*). Standardized coefficients (*β*) are reported in the text for interpretative purposes.

BIS-11, Barratt Impulsiveness Scale; TEMPS-A, Temperament Evaluation of Memphis, Pisa, Paris and San Diego Autoquestionnaire; RSA, Resilience Scale for Adults.

*p < 0.05, **p < 0.001.

## 4 Discussion

Resilience refers to the capacity to adapt effectively to stressful or traumatic events and to return to a functional psychological state (Masten, 2001). In contemporary psychiatry, it is conceptualized as a multidimensional construct shaped by temperament and character traits, problem-solving skills, and social support systems (Campbell-Sills et al., 2006). In alcohol Use Disorder (AUD), higher resilience has been associated with lower alcohol consumption and fewer alcohol-related problems (Cusack et al., 2023). However, existing models often fall short in explaining how resilience manifests specifically within AUD populations.

Previous studies suggest that resilience functions as a protective factor in the context of trauma and stress. For example, Wingo et al. (2010) showed that trauma-exposed individuals who remained free of PTSD and depression—operationalized as resilient—exhibited better neurocognitive performance, particularly in nonverbal memory, compared to non-resilient counterparts. Likewise, Enoch (2006) emphasized that in the development of alcoholism, resilience may stem from genetic and environmental factors that buffer vulnerability to stress and early adversity. Taken together, these findings support the view that resilience may act as a protective mechanism in stress-related and addiction-prone conditions, rather than merely representing a general mental health trait.

In the present study, resilience was associated with temperament and impulsivity traits. In particular, depressive and irritable temperaments, as well as non-planning impulsivity, emerged as significant correlates of lower resilience. Cyclothymic, anxious, and depressive temperaments also correlated negatively with resilience, highlighting these traits as potential risk markers in AUD.

Depressive temperament showed negative associations with the “perception of self,” “perception of future,” and “family cohesion” subscales of resilience, consistent with prior studies in clinical and non-clinical samples (Kesebir et al., 2013). These findings suggest a broader transdiagnostic pattern whereby depressive affect may impede emotion regulation, future-oriented thinking, and interpersonal bonding (Dalgleish et al., 2020; Harvey et al., 2004). Irritable temperament was likewise negatively related to multiple resilience dimensions (self-perception, future perception, structured style, and family cohesion), in line with reports from depression and other psychiatric conditions (Cloninger et al., 1993; Kesebir et al., 2013; Rettew and McKee, 2005). Hyperthymic temperament showed a weak positive link with resilience—possibly reflecting optimism, energy, and sociability (Akiskal et al., 1983; Kesebir et al., 2013)—but its overall impact appeared limited in our AUD sample, potentially due to stronger effects of impulsivity.

Among impulsivity dimensions, non-planning impulsivity showed the most consistent negative association with resilience. Individuals high on this trait tend to prioritize immediate rewards and insufficiently consider long-term consequences (Patton et al., 1995). Prior work indicates that non-planning impulsivity is elevated in substance use disorders and is related to relapse and poorer treatment outcomes (Khemiri et al., 2021). In line with this literature, our findings suggest that non-planning impulsivity may undermine resilience in AUD (Hamilton et al., 2014; Sliedrecht et al., 2021). With regard to aggression, aside from a weak negative correlation between hostility and perception of future, no consistent associations were observed—consistent with prior reports of weak or inconsistent links between aggression and resilience in substance-using populations (Şenormanci et al., 2025). These findings may reflect sample characteristics or measurement limitations and warrants further study.

Childhood emotional abuse and neglect were weakly but significantly associated with lower resilience—especially within the “perception of future” domain—indicating that emotional maltreatment may erode hope and future-oriented thinking, which are central elements of intrapersonal resilience. Prior studies have highlighted relatively stronger effects of emotional neglect on resilience compared to other trauma types (Lee et al., 2018). In contrast, physical and sexual abuse did not show significant associations with overall resilience in our sample, which may be attributable to sample-specific characteristics (e.g., all-male population) or underreporting of these experiences. Notably, sexual abuse was weakly negatively associated with the “structured style” subscale, suggesting that individuals with such experiences may have greater difficulty maintaining organized daily routines and behavioral consistency.

Participants without a history of suicide attempts had higher “perception of self” scores, supporting previous findings that stronger self-perception and higher resilience are associated with a lower risk of suicidality (Roy et al., 2007a, 2007b). Resilience dimensions such as self-perception, future orientation, and family cohesion overlap with psychosocial risk and protective factors known to influence suicidality (Beck et al., 2006; Miller et al., 2015). However, few studies have specifically investigated how individual RSA subscales relate to suicidal behavior, highlighting an important direction for future research (Johnson et al., 2011).

From a relapse perspective, individuals with AUD are repeatedly exposed to chronic stress, emotional dysregulation, impulsive decision-making, and a high risk of relapse. In this context, resilience is viewed as a dynamic capacity that supports adaptation and recovery from adversity (Masten, 2014; Fletcher and Sarkar, 2013). Charney (2004) suggests that low resilience reflects impaired stress adaptation, which increases susceptibility to maladaptive coping strategies such as substance use and relapse. Similarly, recent evidence indicates that lower resilience is linked to a heightened risk of relapse in substance use disorders (Yamashita et al., 2021). Within this framework, resilience should not be considered an entirely independent relapse factor; instead, it likely influences relapse risk indirectly by interacting with established factors such as impulsivity, depressive symptoms, and trauma history. In relapse models, self-efficacy—defined as the belief in one’s ability to cope with high-risk situations—is considered a core protective factor (Bandura, 1997; Witkiewitz and Marlatt, 2004). Although resilience and self-efficacy are distinct constructs, resilience encompasses cognitive-emotional resources (e.g., stress tolerance, hope, social support) that can enhance self-efficacy, which in turn is associated with lower relapse risk (Yamashita et al., 2021). Therefore, resilience may function as a higher-order construct that reinforces protective mechanisms such as self-efficacy, rather than replacing established relapse-related factors.

### 4.1 Limitations

The sample consisted only of male participants, which limits the generalizability of the findings. Including female patients might have revealed sex-related differences in resilience, particularly given that women with AUD tend to report higher levels of internalizing symptoms and emotion-driven impulsivity, which could modify the associations between resilience, temperament, and impulsivity. The cross-sectional design prevents causal interpretations. Although *post hoc* power analysis indicated sufficient statistical power for the regression model, the overall sample size was moderate, which may have limited the detection of smaller effect sizes. Additionally, the use of self-report measures may have introduced recall bias and social desirability bias. Finally, although the study followed the three-factor CTQ structure commonly used in Turkish validation research, the lack of a separate physical neglect subscale may restrict comparison with international studies. Moreover, the absence of a healthy control group makes it difficult to determine whether the observed associations are specific to AUD or reflect more general psychological patterns.

## 5 Clinical implications and conclusion

The present study indicates that depressive and irritable temperaments, as well as non-planning impulsivity, are significantly associated with lower resilience in individuals with AUD. Although resilience is influenced by relatively stable personality traits and early-life adversities, it should not be viewed as a fixed characteristic but rather as a dynamic and potentially modifiable capacity. Interventions that focus on enhancing emotion regulation, strengthening future-oriented planning, and improving perceived social support may be particularly beneficial in AUD, as they could bolster resilience and contribute to better treatment adherence and clinical outcomes. Accordingly, early, individualized, and resilience-focused psychosocial interventions may support recovery, reduce relapse vulnerability, and contribute to more favorable clinical trajectories in AUD.

## Data Availability

The raw data supporting the conclusions of this article will be made available by the authors, without undue reservation.
